# Learning from label proportions in brain-computer interfaces: Online unsupervised learning with guarantees

**DOI:** 10.1371/journal.pone.0175856

**Published:** 2017-04-13

**Authors:** David Hübner, Thibault Verhoeven, Konstantin Schmid, Klaus-Robert Müller, Michael Tangermann, Pieter-Jan Kindermans

**Affiliations:** 1 Brain State Decoding Lab, Cluster of Excellence BrainLinks-BrainTools, Department of Computer Science, Albert-Ludwigs-University, Freiburg, Germany; 2 Machine Learning Group, Berlin Institute of Technology, Berlin, Germany; 3 Electronics and Information Systems, Ghent University, Ghent, Belgium; 4 Department of Brain and Cognitive Engineering, Korea University, Seoul, Korea; Centre National de la Recherche Scientifique, FRANCE

## Abstract

**Objective:**

Using traditional approaches, a brain-computer interface (BCI) requires the collection of calibration data for new subjects prior to online use. Calibration time can be reduced or eliminated e.g., by subject-to-subject transfer of a pre-trained classifier or unsupervised adaptive classification methods which learn from scratch and adapt over time. While such heuristics work well in practice, none of them can provide theoretical guarantees. Our objective is to modify an event-related potential (ERP) paradigm to work in unison with the machine learning decoder, and thus to achieve a reliable unsupervised calibrationless decoding with a guarantee to recover the true class means.

**Method:**

We introduce learning from label proportions (LLP) to the BCI community as a new unsupervised, and easy-to-implement classification approach for ERP-based BCIs. The LLP estimates the mean target and non-target responses based on known proportions of these two classes in different groups of the data. We present a visual ERP speller to meet the requirements of LLP. For evaluation, we ran simulations on artificially created data sets and conducted an online BCI study with 13 subjects performing a copy-spelling task.

**Results:**

Theoretical considerations show that LLP is guaranteed to minimize the loss function similar to a corresponding supervised classifier. LLP performed well in simulations and in the online application, where 84.5% of characters were spelled correctly on average without prior calibration.

**Significance:**

The continuously adapting LLP classifier is the first unsupervised decoder for ERP BCIs guaranteed to find the optimal decoder. This makes it an ideal solution to avoid tedious calibration sessions. Additionally, LLP works on complementary principles compared to existing unsupervised methods, opening the door for their further enhancement when combined with LLP.

## Introduction

A brain-computer interface (BCI) is a neurotechnological solution to control a software or a physical device, e.g. allowing physically challenged users to send messages to caregivers or to operate a robotic device without muscular input. In this work, we focus on BCI applications based on event-related potentials (ERPs) measured by electroencephalography (EEG). ERPs are evoked transient brain patterns observed after, but not limited to, external stimulation events. In the field of BCI, the visual highlighting of symbols on a computer screen is the most common stimulus modality [1], but also non-visual stimuli like sounds [2–4], or haptic stimuli [5, 6] are suited for BCI control. By assigning control commands to symbols on a screen, the user can execute a command by focusing attention onto the highlighting events corresponding to the desired symbol. Visual ERP paradigms have been used for different applications, e.g. for spelling [1, 7], web browsing [8], games [7, 9–11], browsing and sharing pictures [12], predicting emergency brakes in a driving scenario [13] and artistic expression through painting [14]. ERP-based BCIs have several desirable features [15]: they are relatively fast, effective for most healthy users [16] and usable for patients [14, 17]. The visual interfaces are easy to grasp and require virtually no subject training. Consequently, ERP-based BCIs are the most widely used BCI paradigms.

The machine learning decoder in ERP paradigms has to discriminate single stimulus events between attended (*target* events), or non attended (*non-target* event). Three reasons make this a very challenging task [18]. First, ERP features of the EEG are obscured by a high noise level and low signal amplitude, resulting in a bad signal-to-noise ratio (SNR). Second, non-stationarities can occur in the data. These are caused by varying factors [19]: motivation, level of attention, fatigue, mental state, learning, changes in contact impedances of EEG electrodes and others. Third, the statistical properties of the ERP signals and the background EEG differ from subject to subject. Thus, subject-specific data is necessary to obtain optimal decoding performance. Unsupervised methods, in contrast to supervised methods, have the benefit of skipping the calibration session to collect labelled data and learn directly on unlabelled data collected during the online use of the decoder [20–23]. This is desirable, because it reduces the preparation time, avoids the problem of potentially measuring wrongly labelled data in the calibration session, e.g. due to mistakes in the communication between instructor and user, and circumvents the difficulty of dealing with changes in the distribution of class-informative ERP features in the data from the calibration phase to the online application. These may occur due to changes in the human-computer interaction [24], e.g. by introducing feedback to the user.

Several attempts have previously been made to reduce or eliminate the calibration time. Transfer learning methods have been successfully introduced where a new experiment of the same subject [25] or a new subject [26–28] can profit from the database of existing subjects. Furthermore, unsupervised adaptation methods are able to drastically decrease the calibration time in ERP paradigms [24, 29] or even completely avoid them [20, 22, 23]. However, the ability to learn from unlabelled examples comes at a price. While these methods generally work well in practice, there is currently no unsupervised algorithm which is guaranteed to converge to the optimal decoder, even if sufficient data is available.

The main contribution of this paper is the introduction of learning from label proportions (LLP) to the field of BCI. LLP is a recently proposed unsupervised classification method [30]. It is capable of learning from unlabelled data and is guaranteed to recover the same target and non-target means as in a scenario where label information would be present. To the best of our knowledge, this is the first unsupervised algorithm for classification of ERP signals that, under the assumption of independent and identically distributed (IID) data points, is guaranteed to find the optimal decoder.

To grasp the main idea of LLP, consider the following scenario. We want to estimate the average weight of men and that of women. We are not able to weigh people individually but are given aggregated data from groups of the population. There are two groups of people. The first one consists of 50 men and 40 women and has a total weight of 6600 kg, the second one comprises 40 men and 60 women and has a total weight of 7100 kg. The avid reader will quickly realize that the average weight can easily be computed by solving a linear system of 2 equations yielding a men’s average weight of 80 kg and a women’s average weight of 65 kg. Surprisingly, the mean weight for men and women can be computed without actually knowing the weight of a single individual man or woman. This yields an unsupervised method where label information is not required. It is sufficient to know the group-wise means and the proportional presence of each class—man and woman—in the different groups. In a similar fashion to the example above, LLP can be applied to an ERP paradigm to reconstruct the mean target and non-target ERP responses which can then be used to train a classifier and classify individual stimuli.

In the following sections, the LLP algorithm will be discussed. First, the theoretical background and properties will be derived in the methods section. Second, the basic LLP principle is simulated on different artificially generated BCI data sets to study the effect of the target and non-target proportions per group on the classification performance. However, as certain assumptions cannot be tested with simulations, the outcome of an online study with 13 subjects will be presented as a proof of concept. The subjects performed a copy-spelling task where the unsupervised LLP classifier was unaware of the labels—these were solely used for a later performance evaluation. Additionally, the performance will be compared to the unsupervised EM-algorithm by Kindermans et al. [22], and possible improvements and application scenarios will be discussed.

## Materials and methods

### Learning from label proportions

#### Theoretical motivation

In supervised binary classification, the goal is to discriminate between two classes. In machine learning, we optimize a loss function depending on the data **x**_*i*_ and the labels *y*_*i*_ ∈ {−1, 1} where *i* = 1…*N* denotes the different samples. In the following, we will assume that a linear classifier *f*(**x**) = **w**^*T*^ **x** is used which assigns a sample **x**_*i*_ to class 1 if *f*(**x**_*i*_) ≥ 0 and to class 2 if *f*(**x**_*i*_) < 0. Because the classification loss cannot be optimized directly since it is discrete and non-convex, machine learning methods optimise a surrogate loss function instead. For a specific subset of losses called *symmetric proper scoring losses* which include the logistic loss and the square loss, we can rewrite the loss function in a form that depends only on the class means and the input data [31]. Below, we have rewritten the square loss as an explicit example.

$$\begin{array}{c} \sum_{i=1}^{N}{\left(w^{T}x_{i}-y_{i}\right)}^{2}=\sum_{i=1}^{N}\left({\left(w^{T}x_{i}\right)}^{2}+1\right)-2w^{T}\left(\sum_{i_{+}}x_{i_{+}}-\sum_{i_{-}}x_{i_{-}}\right) \end{array}$$ (1)

Here *i*_+_ denotes samples from the class with label +1 and *i*_−_ denotes samples from the class with label −1. It is clear that the term $\sum_{i=1}^{N}\left({\left(w^{T}x_{i}\right)}^{2}+1\right)$ does not depend on label information. The second term can now be reformulated as

$$\begin{array}{c} 2w^{T}\left(\sum_{i_{+}}x_{i_{+}}-\sum_{i_{-}}x_{i_{-}}\right)=2w^{T}\left(N_{+}\mu_{+}-N_{-}\mu_{-}\right) \end{array}$$ (2)

where ***μ***_+_ and ***μ***_−_ indicate the average feature vector of the positive class and negative class, respectively. *N*_+_ and *N*_−_ represent the number of samples in each class. From this equation, it becomes clear that the optimization problem can be solved by merely *knowing the class means and number of samples per class* without explicit label information. In the following sections, we will explain how the mean map algorithm [30] for learning from label proportions can be used to estimate these means.

#### Main concept

Consider a two-class problem and G groups of data where each group is a mixture of these two classes with known mixture ratios contained in **Π**. The means of the feature vectors in the groups ***μ***_1_,***μ***_2_, …,***μ***_*G*_ can then be expressed as a function of the class means ***μ***_+_, ***μ***_−_ as follows.

$$\begin{array}{c} \left[\begin{array}{c} \mu_{1}\\ \vdots \\ \mu_{G} \end{array}\right]=\Pi \left[\begin{array}{c} \mu_{+}\\ \mu_{-} \end{array}\right],\;\Pi :=\left[\begin{array}{cc} \pi_{+}^{1} & \pi_{-}^{1}\\ \vdots & \vdots \\ \pi_{+}^{G} & \pi_{-}^{G} \end{array}\right] \end{array}$$ (3)

To obtain an empirical estimate of the group means ***μ***_1_,***μ***_2_, …,***μ***_*G*_, we do not need label information. These quantities can then be used to approximate the class means by using the pseudoinverse of Π, given by $\Pi^{-1}:={\left(\Pi^{T}\Pi \right)}^{-1}\Pi^{T}$. Using this notation, the class means can then be computed as

$$\begin{array}{c} \left[\begin{array}{c} \mu_{+}\\ \mu_{-} \end{array}\right]=\Pi^{-1}\left[\begin{array}{c} \mu_{1}\\ \vdots \\ \mu_{G} \end{array}\right]. \end{array}$$ (4)

Hence, by solving the resulting system of linear equations, we can get an estimation ${\tilde{\mu }}_{+},{\tilde{\mu }}_{-}$ of the true class means ***μ***_+_,***μ***_−_. In the BCI application, these two classes are target and non-target. The implicit *homogeneity assumption* in this formulation is that ***μ***_+_,***μ***_−_ are the same for each group, i.e. the feature distributions for target and non-target samples are independent of the group.

#### Guaranteed convergence

Let $x_{i}^{j}$ denote the j-th feature of **x**_*i*_. If we assume that each feature $x_{1}^{j},x_{2}^{j},\ldots ,x_{N}^{j}$ is drawn independently from an identical distribution (IID) with finite expected value *μ*^*j*^ and variance *σ*, then the central limit theorem (CLT) states that the sample average ${\hat{\mu }}^{j}$ is normally distributed for large *N*.

$$\begin{array}{c} {\hat{\mu }}^{j}:=\frac{x_{1}^{j}+\ldots +x_{N}^{j}}{N}∼N\left(\mu^{j},\frac{\sigma^{2}}{N}\right) \end{array}$$ (5)

This implies that, given enough data, the approximations ${\tilde{\mu }}_{1},\ldots ,{\tilde{\mu }}_{G}$ converge to ***μ***_1_, …,***μ***_*G*_ in our scenario. After solving the linear system, we therefore have an estimation of the class-wise means which is *guaranteed to converge* for *N* → ∞. Hence, we have an unsupervised classifier that, under the assumption of IID and homogeneity, is guaranteed to minimize the squared error loss. Additionally, there also exists a version of the mean-map algorithm using a manifold regulariser that performs better than this version under a violation of the homogeneity assumption [31].

#### Noise amplification factor

Like other unsupervised algorithms, LLP performs worse compared to a supervised classifier when only a limited number of data points is available. In the LLP case, we can directly quantify the difference which depends on the number of groups (*G*) and the inverse of the mixture matrix **Π**^−1^. Note that we use the pseudo-inverse if *G* > 2 and denote it by

$$\begin{array}{c} \Pi^{-1}:=\left[\begin{array}{ccc} \nu_{+}^{1} & \ldots & \nu_{+}^{G}\\ \nu_{-}^{2} & \ldots & \nu_{-}^{G} \end{array}\right]. \end{array}$$ (6)

Now using the properties of the variance and Eq 4, the variance of one feature $\mu_{k}^{j}$ of the positive class means ***μ***_+_ can be computed for each feature *j* as

$$\begin{array}{c} \mathrm{Var}\left({\hat{\mu }}_{+}^{j}\right)=\mathrm{Var}\left(\sum_{k=1}^{G}\nu_{+}^{k}{\hat{\mu }}_{k}^{j}\right)=\sum_{k=1}^{G}{\left(\nu_{+}^{k}\right)}^{2}\mathrm{Var}\left({\hat{\mu }}_{k}^{j}\right) \end{array}$$ (7)

and analogously for features of the negative class ***μ***_−_. This implies that the variance of each mean is amplified by the square of the pseudoinverse coefficients. If the features are normally distributed, which is closely followed by neural control signals in BCI [32], and we make the additional assumption, that all features are IID with the same variance *σ*, then the variance of each group mean feature is given by

$$\begin{array}{c} \mathrm{Var}\left({\hat{\mu }}_{k}^{j}\right)=\frac{\sigma^{2}}{\frac{N}{G}},\;k=1\ldots G \end{array}$$ (8)

where $\frac{N}{G}$ is the fraction of data available per group. To quantify the increased variance of the class-wise mean estimation compared to the original variance of $\frac{\sigma^{2}}{N}$, we define the *noise amplification factor* (NAF) as the number of groups G multiplied with the squared Frobenius norm, which is the sum of all squared coefficients in **Π**^−1^.

$$\begin{array}{c} \mathrm{NAF}≔G\cdot \sum_{c\in \{+,-\}}\;\sum_{k=1}^{G}{\left(\nu_{c}^{k}\right)}^{2} \end{array}$$ (9)

The NAF calculates the increased amount of data needed for LLP to obtain a similar mean estimation performance as a supervised method. In the result section, we will evaluate the performance for different numbers of groups and mixture matrices to see to which extent this theory matches actual performance results.

### Sequence generation and spelling interface

Now, we address how these different groups of data can be obtained. We propose to tune the stimulus presentation to maximize the power of the machine learning algorithm. To achieve this symbiosis, we started from the original visual ERP speller [1] and did several modifications. First, an additional column was added to increase the total number of symbols resulting in a 6 × 7 grid. We included all letters of the alphabet plus the symbols “⎵” “.” “,” “!” “?” “←” and 10 “#” symbols which account for visual blanks. The meaning of those is explained further below.

To generate two different groups of data (*G* = 2) in the visual speller, we used the stimulus presentation paradigm created by Verhoeven et al. [33]. This paradigm is flexible in the sense that it can generate sequences with a desired mixture ratio of target and non-target stimuli. At the same time, it uses a heuristic to increase the signal-to-noise ratio in the stimulus responses by avoiding the two most common spelling errors: adjacency distraction and double flashes. Our modification requires two distinct sequences with differing target to non-target ratios—as these ratios form the label proportions exploited by LLP.

Stimuli from sequence 1 highlight each character exactly 3 times for every 8 stimuli. This means that no matter which character the user is focussing on, we obtain 3 targets and 5 non-targets in this train of 8 stimuli. However, the decoding method and sequence generator are unaware about the exact target positions, i.e., where the 3 targets are located within these 8 stimuli. Similarly, sequence 2 contains trains of 18 highlighting events where each character is only highlighted twice. This leads to a ratio of 2 targets and 16 non-targets out of 18 stimuli in the second sequence These known target and non-target ratios are now exploited by writing the sequence-wise means ***μ***_1_ and ***μ***_2_ as a function of the mean target ***μ***_+_ and non-target ERP responses ***μ***_−_.

μ1=138μ++58μ-μ2=218μ++1618μ- (10)

For this simple configuration, the mean target and non-target ERP responses can be computed directly by solving the linear system yielding the following two equations.

μ+=-3.37μ1-2.37μ2μ-=-0.42μ1+1.42μ2 (11)

In ERP terminology, a trial corresponds to the selection of a single command or spelling a single character. In our approach, a trial consisted of 4 sequences of length 8 with 3 targets each and 2 sequences of length 18 with 2 targets each, totalling to 68 highlighting events. The 16 targets and 52 non-targets highlighted per trial each resulted in an ERP response in the EEG, leading to 68 ERP *epochs*.

A few additional measures were taken to comply with our assumption that ERP responses are distributed identically and homogeneously within each group. First of all, it is known that the response upon a stimulus event is influenced by its brightness and thus by the number of symbols highlighted within that stimulus event [34]. To equalize the number of highlighted symbols among stimuli, the 10 “#” symbols were introduced in addition to the standard symbols. Adding them balances the brightness of those stimuli containing less informative symbols otherwise. As they never convey information, they take the role of non-target symbols, and do not alter the mixture ratios of the sequences. They are only highlighted in sequence 2 to obtain the high non-target ratio while highlighting a total of 12 symbols per epoch—the same number as in sequence 1. The second precaution we took, is to have sequences from both groups randomly interleaved within a trial. This avoids violating the homogeneity assumption, e.g., non-stationarity in the feature distribution within one trial or a modulation of the P300 amplitude because of differences in the target-to-target interval [35].

**Fig 1:**
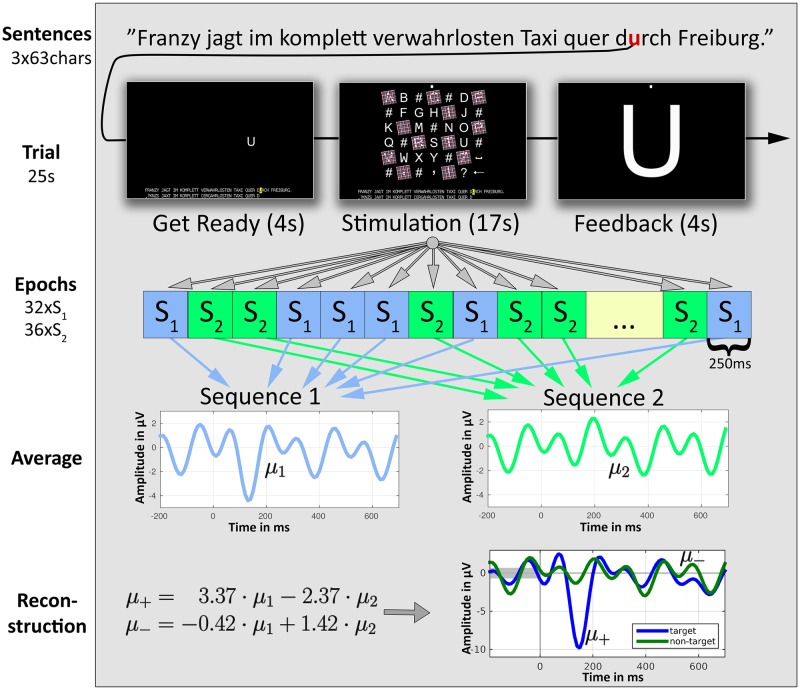
Scheme of the experimental structure and LLP classifier. Top to bottom: The sentence “Franzy jagt …” is spelled three times. To spell a single character in one trial, 68 highlighting events occur, with 32 belonging to sequence 1 and 36 belonging to sequence 2. The resulting 68 ERP responses (epochs) are averaged for each sequence, and these averages are exploited to reconstruct the mean target and non-target ERP responses.

For stimulus presentation, a salient highlighting method proposed by Tangermann et al. [12] was implemented. It uses a combination of brightness enhancement, rotation, enlargement and a trichromatic grid overlay. An example of the highlighting scheme in the spelling matrix is shown in Fig 1.

### Experimental protocol, data quantity and task timing

The subjects were asked to spell the sentence: “Franzy jagt im komplett verwahrlosten Taxi quer durch Freiburg”. The sentence was chosen because it contains each letter used in German at least once. Each subject spelled this sentence three times. The stimulus onset asynchrony (SOA) was 250 ms (corresponding to 15 frames on the LCD screen utilized) while the stimulus duration was 100 ms (corresponding to 6 frames on the LCD screen utilized). For each character, 68 highlighting events occurred and a total of 63 characters were spelled three times. This resulted in a total of 68 ⋅ 63 ⋅ 3 = 12852 EEG epochs per subject. Spelling one character took around 25 s including 4 s for cueing the current symbol, 17 s for highlighting and 4 s to provide feedback to the user. Assuming a perfect decoding, these timing constraints would allow for a maximum spelling speed of 2.4 characters per minute. Fig 1 shows the complete experimental structure and how LLP is used to reconstruct average target and non-target ERP responses.

### Subjects and ethics

Overall, 13 subjects (5 female, average age: 26 years, std: 1.5 years) were recruited. Only one subject (S2) had prior EEG experience. The EEG study was approved by the Ethics Committee of the University Medical Center Freiburg. Following the principles of the Declaration of Helsinki, written informed consent was obtained from the subjects prior to participation. One session took about 3 hours (including EEG set-up and washing the hair), and participants were compensated with 8 Euros per hour.

### EEG data acquisition

Subjects were placed in a chair at 80 cm distance from a 24-inch flat screen. EEG signals from 31 passive Ag/AgCl electrodes (EasyCap) were recorded, which were placed approximately equidistantly according to the extended 10–20 system, and whose impedances were kept below 20 kΩ. All channels were referenced against the nose and the ground was at FCz. The signals were registered by multichannel EEG amplifiers (BrainAmp DC, Brain Products) at a sampling rate of 1 kHz. To control for vertical ocular movements and eye blinks, we recorded with an EOG electrode placed below the right eye and referenced against the EEG channel Fp2 above the eye. In addition, pulse and breathing activity were recorded. However, the EOG, pulse and breathing signals did not enter the further analysis. Markers obtained from an optical sensor on the screen indicated the exact starting time point of each highlighting event.

The data of all 13 subjects together with the data used for the simulations (see below) is freely available online at http://doi.org/10.5281/zenodo.192684.

### Preprocessing, classification and scoring

#### Preprocessing

To process the data in the online experiment and during offline re-analysis, the BBCI Toolbox was used [7]. In both cases, the collected data was bandpass filtered with a third order Chebyshev Type II filter between 0.5 and 8 Hz and downsampled to 100 Hz. Epochs were windowed to [-200, 700] ms relative to the stimulus onset and corrected for baseline shifts observed in the interval [-200, 0] ms. After dismissing channels Fp1 and Fp2, features describing the elicited transient potentials were extracted from the remaining 29 EEG channels. Per channel, the mean amplitudes of six intervals ([50, 120], [121, 200], [201, 280], [281, 380], [381, 530] and [531, 700] ms) were computed, resulting in a representation of each epoch by 6 ⋅ 29 = 174 features.

#### Classification

At the end of each trial, the LLP algorithm was applied on the complete set of observed responses in order to estimate the class means ***μ***_+_ and ***μ***_−_. Additionally, the pooled (global) covariance matrix **Σ** on combined data of both classes was estimated using shrinkage-regularization as initially proposed by Ledoit&Wolf [36] and first applied in BCI by Vidaurre et al. [37], see also [32]. The shrinkage parameter was chosen automatically using the Ledoit—Wolf formula [38]. Based on the reconstructed class means and the pooled covariance matrix, the projection vector **w** was computed as

$$\begin{array}{c} w=\Sigma^{-1}\left(\mu_{+}-\mu_{-}\right) \end{array}$$ (12)

and applied to the features of a new epoch **x**_**new**_ as *f*(**x**_**new**_) = **w**^**T**^ **x**_**new**_. Technically, this can be understood as a least squares classifier with re-scaled outputs and is known to lead to the same direction of the projection vector given the correctly recovered class means [39]. To select a symbol in each trial, classifier outputs were summed up for each symbol and the symbol with the highest sum was chosen. Note that this decision does not depend on the bias term, because the same bias is summed up for each symbol and thus, its effect cancels out when taking the maximum. Visual blanks (’#’) were excluded from being chosen as selected symbols.

The classifier was reset and started from scratch for each of the three spellings of the sentence “Franzy jagt …” in the online experiment. After collecting the data of a new character, the classifier was retrained. Label information (target / non-target role of characters) were used exclusively to evaluate the performance during offline analyses, but never to train the LLP classifier or generate the sequences during online use.

In the supervised scenario, which was used solely in the offline analysis for comparison, class-wise means and the class-wise covariance matrices were computed based on label information using the sample statistics. Shrinkage-regularization and the projection vector were computed as described before. We refer to this classifier as shrinkage-LDA [32].

#### Scoring

To assess the performance of any classifier, the area under the curve (AUC) of the receiver-operator characteristics curve (ROC) for classifying target vs. non-target epochs was calculated. The AUC values can range between 0 and 1, with a theoretical chance level of 0.5. An AUC value of 1 indicates perfect separation between the two classes. The AUC can be seen as the probability that the output of the LDA ranks a target higher than a non-target. We chose AUC as it is non-parametric and independent of a classifier bias. When we report an AUC, it always considers the binary target vs. non-target classification task. In addition to the AUC metric, the percentage of correctly classified symbols is reported where appropriate to describe the performance in the online spelling application. Please note, that because we did not aim to maximize the spelling speed in this study and because the assumptions required to use the information transfer rate (ITR) reliably are not met by ERP paradigms [40], we did not report bit-rates in this study.

### Unsupervised post hoc classification

An interesting feature of adaptive classifiers is that their quality improves over time as more and more unlabelled data becomes available during their online application. Hence, re-analysing previous trials may result in more accurate decoding results compared to the results obtained online. This so-called post hoc re-analysis can easily be included in an online experiment as done before by Kindermans et al. [22]. In applications like text spelling, the constant post hoc re-analysis may prove extremely beneficial to correct early spelling mistakes, at the start of the spelling procedure, when data is still scarce. In a real-life spelling task, the user would need to accept that early characters initially are misspelled by the system, but would probably be corrected at a later time point. Thus, for the user, it is a fruitful strategy to continue spelling the sentence despite of potential incorrectly decoded characters.

### Artificial data sets for simulations

In addition to data collected during an online experiment, we created artificial data sets to quantitatively assess whether a lower noise amplification factor (NAF) leads to a better performance as suggested in our theoretical considerations. The artificial sequences were based on EEG data of two real ERP-based BCI data sets. The first data set stemmed from a visual attention task with 6 possible choices, whereas the second data set had been recorded with an auditory ERP paradigm with spatial cues similar to the AMUSE paradigm [2]. Both data sets had been recorded under an SOA of 250 ms. In both paradigms, 4860 epochs were recorded with the same 5 young healthy volunteers each. These data sets were chosen for simulations in order to cover different SNR values—the first data set has a very high SNR, while the auditory data set displays a low SNR compared to data obtained from visual ERP paradigms. For each data set, artificial sequences were created by assigning target and non-target epochs randomly to each of the new sequences based on a pre-defined mixture matrix **Π**. This was done for different mixture matrices and a varying number of epochs ranging from 500 to 4860 where epochs were taken chronologically starting from the beginning of the experiment. For each of the mixing matrices and number of epochs, an LLP classifier based on the reconstructed means and the pooled covariance matrix was trained and tested together with a supervised classifier in a 5-fold chronological cross-validation. In contrast to our LLP online study, 64 channels were used, eye-artefacts were regressed out using the EOG channel [41] and the intervals [100 180], [181 300], [301 400], [401 600], [601 850] and [851 1200] ms were used in case of the auditory study. The other pre-processing steps are the same as mentioned above.

### Bootstrapping

A leave-one-out bootstrapping test was performed offline to assess whether the homogeneity assumption holds for the data recorded in the online experiment. The idea is to compute the similarity of a sample from sequence 1 to the average ERP response from sequence 1 and to the average ERP response from sequence 2. The similarity values allow testing whether the null hypothesis holds that target and non-target responses follow the same distribution for both sequences. After applying the same preprocessing steps as mentioned before, we iterated over each target (non-target) epoch of sequence 1. The average target (non-target) ERP responses for both sequences were computed where the selected epoch was excluded when calculating the average of sequence 1. In the next step, the squared distance (*L*_2_—norm) between the selected epoch and the previously computed averages was calculated in the interval [0, 700] ms using all channels. A two-sided T-test was finally conducted to check, whether these distances differ significantly. This procedure was done separately for each class and subject, yielding a total of 2 ⋅ 13 = 26 tests.

## Results

The result section is divided into three parts. First, we present the simulation results, subsequently the online study is evaluated and finally a comparison between LLP and the EM-algorithm by Kindermans et al. [21–23] is presented.

### Simulations

**Fig 2:**
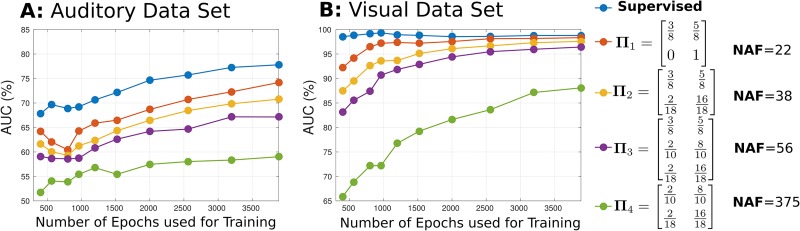
Classification results of LLP applied on artificial data sets generated from an auditory (A) and a visual (B) ERP paradigm. For each artificial data set, the target vs non-target accuracy result for a supervised shrinkage-LDA and different mixing matrices **Π**_1_–**Π**_4_ is shown. The first column in the matrices always denotes the target ratios while the second column denotes the non-target ratio. NAF = noise amplification factor.

To evaluate the feasibility of LLP for BCI and to validate the theoretical considerations, we performed simulations on artificial data sets generated as described before. Creating artificial data sets allowed us to assess the effect of the mixing matrix on the quality of the class-wise mean reconstruction. Four different mixing matrices were used, ranging from one with extremely different target and non-target mixture ratios (**Π**_1_) to one with relatively similar sequences (**Π**_4_). The following observations can be made from the simulation results in Fig 2. First, the classifier performs well above chance level (50%) on the auditory and visual data sets, indicating LLPs feasibility to reconstruct the class means. As expected, classification accuracy is much higher on the visual data (Fig 2B) where the algorithm reaches almost perfect performance for the well-conditioned mixing matrices **Π**_1_–**Π**_3_. The performance on the auditory data set is worse (Fig 2A), but still exceeds an AUC of 70% for matrices **Π**_1_ and **Π**_2_ when provided with a sufficient amount of data. Second, we observe, that the performance over the four matrices can be ranked in the order of the ascending noise amplification factors, indicating that the NAF is a good parameter to characterize how well a mixing matrix determines the LLP performance. Third, we observe that the learning curve of LLP resembles a supervised method, however, a substantial amount of additional data is required to reach the same performance level. Finally, we observed that mixing matrix **Π**_3_ with three different sequences performs worse than some of the mixing matrices with two sequences. A reduction to only two out of three sequences, namely the one with the highest target ratio and the one with the highest non-target ratio, seems to be preferable over maintaining all three sequences, e.g. dropping sequence [2/10, 8/10] from **Π**_3_ yields **Π**_2_, which has a lower NAF and a higher performance.

The results obtained from the offline simulations indicate the feasibility to use LLP on data of ERP-based BCIs. However, the central homogeneity assumption, i.e. that target and non-target ERP responses follow the same distributions for all sequences, could not be tested in simulations. Hence, there is a need for an online study which we conducted with 13 subjects.

### Online experiment

#### Basic neurophysiology and supervised performance

**Fig 3:**
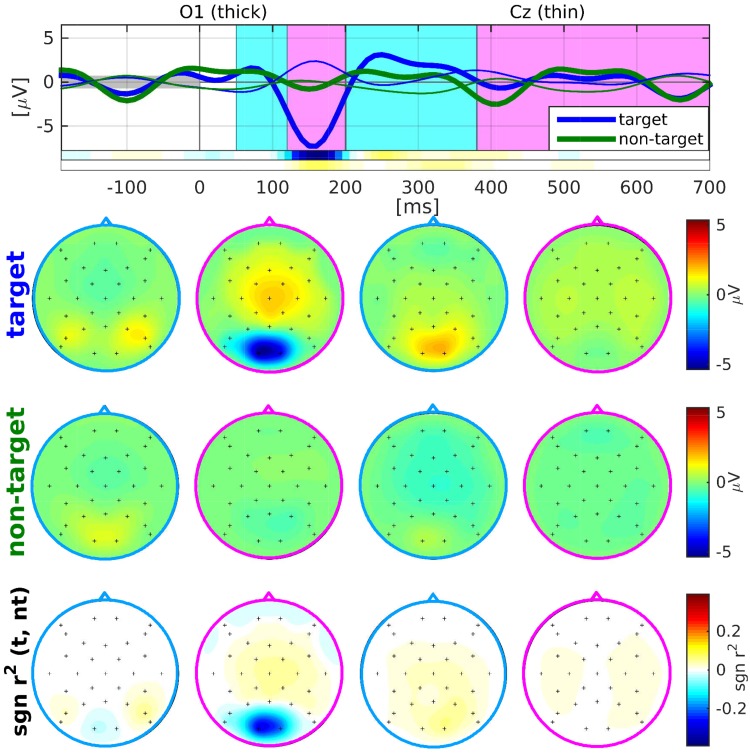
Grand average (N = 13) visual ERP response. Top row: Average responses evoked by visual target (blue) and non-target (green) stimuli in the occipital channel O1 (thick) and the central channel Cz (thin) during the online experiment. Prior to averaging, a baseline correction was performed based on data within the interval [-200, 0] ms. The signed *R*^2^ values for channels O1 and Cz over time are provided by two horizontal colour bars. Their scale is identical to the scale of the plots in the bottom row of scalp plots. Middle rows: Scalp plots visualising the spatial distribution of mean target and non-target responses within four selected time intervals: [50 120], [120 200], [201 380] and [381 700] ms relative to stimulus onset. Bottom row: Scalp plots with signed *R*^2^ values indicate spatial areas with high class-discriminative information.

First, we inspected the class-wise visual ERP responses to assess the quality of the data of the online study. They are provided as grand average responses in Fig 3. The rhythmic characteristic of the non-target responses generally reflects the SOA of 250 ms. We found a strong early negative ERP upon target stimuli over the occipital lobe (hereafter called N150) at around 150 ms for almost all subjects with an average amplitude of around −8*μV*. For non-target stimuli, the N150 was very reduced. The late positivity of targets (hereafter called P300) in the central electrodes is rather late and weak with an average peak time around 400 ms and an average amplitude of only around 2*μV*. Table 1 lists the amplitudes and peak latencies per subject observed for channels O1 (for the N150) and Cz (for the P300).

**Table 1:** Overview of neurophysiological features and supervised classification performance.

Subject	N150 (O1)		P300 (Cz)		AUC (%)
	*Ampl*. (*μV*)	*Lat*. (*ms*)	*Ampl*. (*μV*)	*Lat*. (*ms*)	
S1	−9.76	150	2.72	340	98.85
S2	−11.11	150	1.48	400	98.73
S3	−5.63	170	1.94	500	98.06
S4	−9.48	160	−0.25	500	99.82
S5	−7.59	160	1.15	410	97.05
S6	−12.17	170	0.65	470	97.12
S7	−7.79	150	1.13	450	99.92
S8	−3.57	180	3.87	360	91.69
S9	−13.25	140	0.11	380	99.56
S10	−12.01	140	3.67	380	99.72
S11	−2.93	180	1.31	300	89.18
S12	−4.35	150	3.49	370	98.89
S13	−4.10	160	3.57	370	98.45
**Mean**	−**7.98**	**158.46**	**1.91**	**402.31**	**97.46**

The amplitude and latency of peak amplitudes were derived after epoch-wise baseline removal and class-wise averaging of epochs. Values reported for N150 were determined as the minimum of channel O1 of the interval [100 200] ms, while the late positivity (P300) was derived as the maximum of channel Cz in the interval [250 500] ms. The last column lists the AUC values estimated via cross-validation from a supervised classifier (see text).

By training a supervised shrinkage-LDA on this data set in an offline analysis and calculating the binary target vs. non-target classification accuracy based on a 5-fold chronological cross-validation, we obtained an average AUC of 97.5% which indicates a very good SNR of the data set. Note that only the few preprocessing steps mentioned in the preprocessing method section were applied and no artefact removal or adjustment of the classification time intervals was performed.

#### Homogeneity

**Fig 4:**
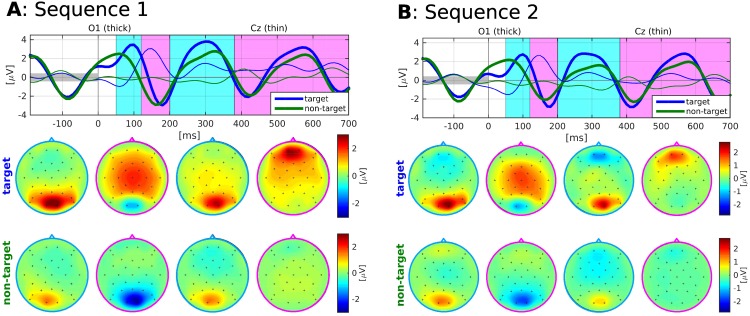
ERP responses for S11 of sequence 1 (A) and sequence 2 (B). Top row: Average responses evoked by target (blue) and non-target (green) stimuli in the occipital channel O1 (thick) and the central channel Cz (thin). Prior to averaging, a baseline correction was performed based on data within the interval [-200, 0] ms. Bottom rows: Scalp plots visualising the spatial distribution of mean target and non-target responses within four selected time intervals: [50 120], [120 200], [201 380] and [381 700] ms.

To test the homogeneity assumptions of LLP, i.e. that both sequences have the same average target and non-target ERP responses, we visually inspected the responses for both sequences and each subject with the goal to detect systematic differences in the ERP amplitudes and latencies between the two sequences. Fig 4 shows the ERP plots from subject S11 for both sequences. Even though small differences can be observed, the ERP responses generally look extremely similar and we could not detect any systematic differences by visual inspection. We also performed a bootstrapping test, as explained in the method section, comparing the similarity of a sample from sequence 1 to the average ERP responses of both sequences. The significance level was corrected by dividing by 13 accounting for testing on 13 independent subject. One significant difference for the corrected significance level (p* = 0.05/13) was found, namely the differences in target ERP responses for S4. We will later see that subject S4 nevertheless performed well.

#### Reconstructed means

**Fig 5:**
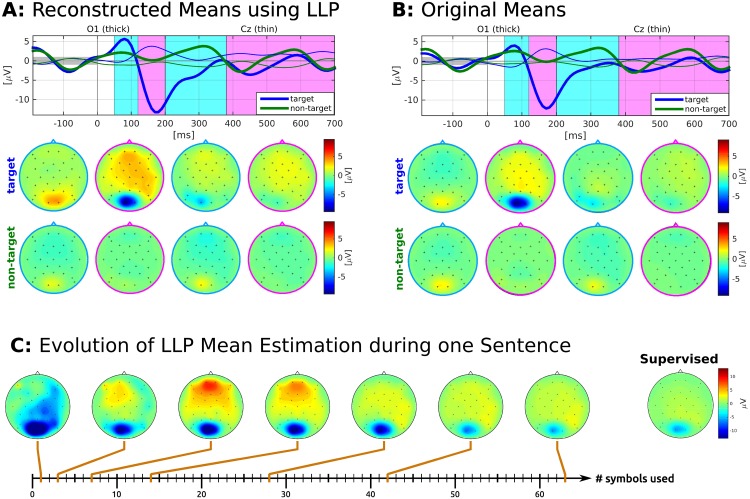
ERP responses for S6 of the reconstructed class-wise means using LLP (A) and original labelled data (B). C shows the LLP target estimations in [120 200] ms for different numbers of training points. For details, see description of Fig 4.

Next, we investigated if LLP could correctly reconstruct the mean target and non-target ERP responses, when the full amount of data corresponding to three sentences is provided. The ERP plots for subject S6 and four intervals are given in Fig 5. It compares the target and non-target ERP means estimated by LLP (Fig 5A) with the true class means (Fig 5B). We observe, that the reconstructed class means capture the characteristics of the original means almost flawlessly.

It is also of interest, how the class means estimated by LLP evolve using a growing amount of data. As an example the target mean for subject S6 is provided in Fig 5C. Using epochs that correspond to 1, 3, 7, 14, 28, 42 and 63 symbols, the mean target pattern in the interval [120 200] ms stabilizes towards the supervised true mean. While the negative potential over occipital channels undergoes a linear development from strong to weak intensity, the activity in frontal and central channels reveals jumps between negative and positive potentials specifically during the first 10 symbols until finally converging towards the ground truth.

#### Online spelling performance

**Fig 6:**
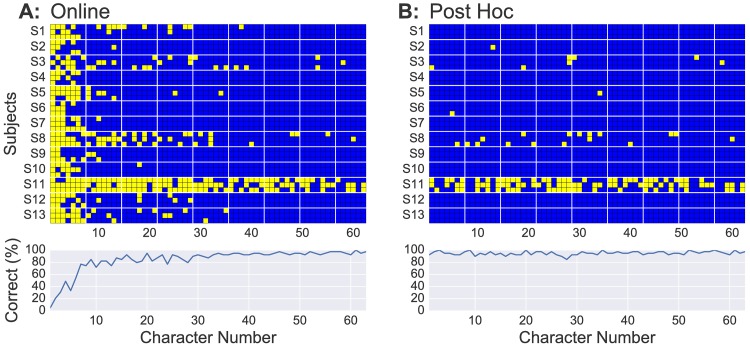
Spelling performance as seen online using the LLP (A) and after the post hoc re-analysis with LLP (B). Top: Each row represents a single spelling of the test sentence “Franzy jagt …”, with yellow squares indicating incorrectly spelled characters and blue squares indicating correctly spelled characters. Bottom: The averaged spelling accuracy across sentences and subjects is shown for each character.

Fig 6A shows the character-wise online spelling performance with LLP for all 13 subjects including the grand average. In total, 84.5% of all characters were spelled correctly (chance level = 3%). After a ramp-up phase of around 7 characters (which corresponds to 3 minutes wall clock time), this accuracy reaches 90.2% correct characters on the remaining characters on average. In general, the algorithm worked well for all subjects except for S11. The reason for S11’s low performance could be determined as an overall low SNR. It is evident also when looking at the supervised performance values provided in Table 1 and by the lack of class-discriminative N150 depicted by Fig 4. However, we could not observe that the data of S11 explicitly violated the homogeneity assumption, see also Fig 4.

As mentioned before, the advantage of an unsupervised adaptive classifier in a spelling application is that early trials can constantly be re-analysed at any later stage of the spelling, when an improved classifier may be available. A re-evaluation of all characters with the classifier obtained at the end of each sentence is provided by Fig 6B. The post hoc performance of the LLP is extremely high, showing zero or one error for 10 out of 13 subjects. The post hoc classifier is able to resolve the majority of characters misclassified by the online LLP seen in Fig 6A.

### Comparing LLP to another unsupervised algorithm

In the previous section, we showed that LLP can be used successfully as a novel classification method in an online study. Given the high SNR of the visual ERP data in the online study, the question remains how well LLP performs in comparison to other unsupervised methods. We chose to compare LLP with another unsupervised algorithm in ERP-based BCI which was successfully used in online classification without prior training, namely the unsupervised classification approach based on expectation-maximization (EM) by Kindermans et al. [22]. The EM algorithm makes use of a probabilistic model that describes the ERP decoding. Although it has no guarantees to converge to a good classifier, it works well in practice when several randomly initialised classifiers are used in parallel.

The comparison between the LLP- and EM-based unsupervised approaches was done using the data set obtained from our online study. To provide a fair comparison of both classifiers, we simulated an online scenario where both classifiers were retrained after each character and were reset to a random initial state before each sentence. The performance was evaluated on the training set and made use of the label information. Note that over-fitting is less an issue for the two approaches, since no label information are used for training any of the two classifiers. For the EM algorithm, we used the same parameters as described in [22].

**Fig 7:**
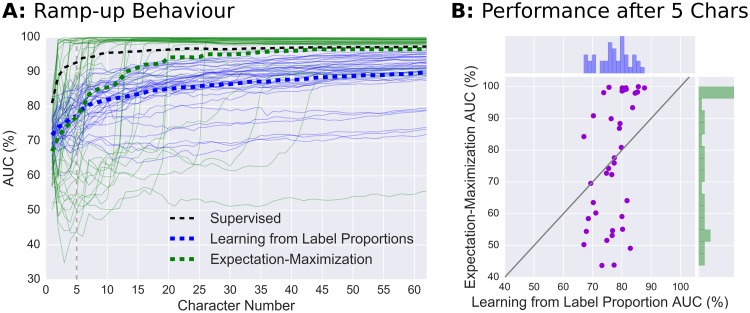
Comparison of LLP to an unsupervised EM-based classification approach for each sentence (A) and after 5 characters (B). **A**: Thin lines represent the binary target vs. non-target AUC performance of the two learning models with every line corresponding to the spelling of a single sentence. Each of the subjects (*N* = 13) spelled each sentence three times resulting in 39 lines. Dashed lines depict average performances. Please note that the supervised shrinkage-LDA was trained and tested in a 5-fold crossvalidation to avoid overfitting. This means that the supervised method only had 80% of the data compared to the unsupervised methods. **B**: Each dot represents the EM and LLP performance after 5 characters for the same subject and sentence.

The ramp-up performance curves of both classifiers in each of the spelled sentences are depicted in Fig 7A. A comparison of the curve shapes indicates that the two algorithms work in different ways and exploit information contained in the data in different ways. While LLP is constantly improving over time and performs well above chance level for all sentences, the EM algorithm behaves dichotomous: depending on the initialisation, it either works extremely well from an early time point on, or fails to display significant performance increases for a relatively long time period. This dichotomous behaviour can also be seen in Fig 7B where the performance comparison of both classifiers after 5 characters is shown. One can see that the LLP performance for each subject and sentence is between 65% and 90% whereas the EM performance is very spread out with instances below chance level (50%) and cases with almost perfect performance. After having learned on the full data of 63 characters, the EM-based approach outperforms LLP on most sentences almost reaching the performance of a supervised classifier.

## Discussion

We have shown how a visual ERP speller can be adapted to meet the prerequisites of LLP. This yields a classifier with not only low conceptual and computational complexity, but also with a guarantee to recover the correct class means without using label information. To the best of our knowledge, none of the existing unsupervised classifiers have this guarantee. The main contribution of this work is to introduce the LLP concept to the BCI community and show that the homogeneity assumption generally holds in an online visual ERP spelling application when using our proposed alterations. However, the goal of this work was not to optimize information transfer per time. Hence, the spelling speed with a maximum number of 2.4 characters per minute is not yet competitive to state-of-the-art unsupervised or supervised methods, even when the calibration time is taken into account. The fastest current supervised speller is based on steady-state visual evoked potential (SSVEP) with around 60 characters per minute [42].

### Possible extensions

We believe that extensions based on the LLP principle are necessary to obtain a competitive unsupervised method. We want to specifically mention two ideas. First, LLP could be combined with other unsupervised methods, e.g., with the minimum distance to Riemannian mean (MDRM) [20] based on distances in the covariance space. There, a prototypical ERP response for one class is necessary and could be obtained via LLP. One could also combine LLP with the EM-algorithm [22]. This is based on the observation that both classifiers outperform each-other during different parts of the experiment. Especially at the start of the ramp-up phase, the probabilistic mean estimation and random initialisation in the EM-algorithm could significantly benefit from the relatively robust mean estimations obtained from LLP. Following this hypothesis, a combined approach could lead to a faster ramp-up behaviour and a more robust classifier compared to the traditional EM-algorithm and outperform the stand-alone LLP especially during the later part of the experiment. Second, LLP could be used in a transfer learning scenario where one starts with a general classifier obtained on several other subjects and utilizes LLP as an unsupervised adaptation method with guarantees. The extreme simplicity of LLP could facilitate both extensions. Changes to the paradigm like reducing the SOA or additional extensions such as artefact removal, early stopping or making use of language models can be expected to further increase the spelling speed [23], but are rather independent of LLP as a classification approach.

### Mixture matrix

Learning from label proportions crucially depends on the possibility to include at least two sequences with different target to non-target ratios into the BCI paradigm. Without this modification, it is not directly applicable to standard ERP paradigms. The best performance can be obtained, when one sequence predominantly contains targets and the other sequence predominantly contains non-targets. (In the limit, this would lead to a supervised scenario.) However, it is important to realize that practical limitations come into play when choosing the sequences. For instance, enforcing a target ratio of 1/2 requires a simultaneous highlighting of half of the selectable symbols, which may be undesired from a usability point of view. If another sequence only consists of non-targets (visual blanks) and the number of highlighted symbols needs to be matched, this would require that half the amount of selectable characters are to be added as visual blanks. This would drastically increase the matrix size. Additionally, if many symbols are highlighted simultaneously, then the number of epochs required to obtain unique decodability of a character increases [33].

In our experiment, we opted for one sequence of stimuli dominated by targets (sequence 1) and one sequence dominated by non-targets (sequence 2). Our specific choice of the sequences and associated mixing matrix reflects a trade-off between classifier quality, spelling matrix size and sequence length. However, other choices are also possible, of course. Future work should experiment with more extreme ratios as they bear the potential of having better performances.

### Visual highlighting scheme

Comparing to previous studies with visual ERPs, the N150 elicited for target stimuli in this online study is very large [43–45], even when compared to using familiar faces as stimuli [46, 47] or motion onset [48]. It may be caused by three factors: First and most importantly, the trichromatic grid overlay is perceived as a very salient stimulus compared to traditional brightness intensifications. The short rotation of the grid may have been beneficial for the saliency as well [48], even though Tangermann et al. [49] found that most of the salience improvement compared to brightness highlighting is caused by the grid effect alone. Second, the SOA of 250 ms is rather long for a visual paradigm. While target-to-target distance is known as a covariate for P300 amplitude [35], longer SOAs may also have an effect on other ERP components. Third, we used precise optical markers to determine stimulus onset time points. Compared to an alternative strategy to use markers elicited by the presentation software, jitter and delay caused by the graphics adapter and the LCD screen are eliminated by the optical markers. This improves the average supervised classification performance by approximately 0.5%.

### Limitations

It is known that the EEG feature distribution can change over the course of a session [19, 22, 24] and thus, violate the IID assumption. To counteract them, an adaptive version of the LLP classifier could be implemented similar to the unsupervised adaptation by Vidaurre et al. [24], which gives a higher weight to more recent data points. Fig 7 showed that LLP is not only slowly converging to the right classifier, it also performs relatively well when little training data is available. Hence, using an adaptive LLP might still be advantageous over other methods, because it reliably approximates the target and non-target means even on limited data. Alternatively, other techniques to compensate for non-stationarities could be employed such as covariate-shift adaptation [50] or stationary subspace analysis [51].

Another downside of the proposed approach is a reduction of the spelling speed as a result of assigning no function to some of the stimuli. This increases the matrix size and the number of highlighted symbols, and reduces the size of each individual symbol on the screen. However, as many factors such as the optimal number of highlighted symbols per stimuli or the optimal matrix size are still unknown, it is hard to quantify this loss. One can also consider a strategy where the LLP initially learns on the extended matrix with ‘#’ symbols and then, once it reached a satisfying performance level, switches to an ordinary spelling matrix.

### Application scenarios

Going beyond visual ERP paradigms, we briefly want to outline how LLP can also be used for other ERP paradigms such as auditory or haptic ones. LLP works in a general setting with multiple selection options when strictly assigning a non-target function to one of the possible options. For instance, in the 6-class auditory AMUSE paradigm [2], one could assign no specific control command to one of the 6 stimuli. Hence, this stimulus would never be attended and always be a non-target. The target proportion for the other stimuli would then be 1 out of 5. This yields two groups with different target to non-target ratios such that LLP can be applied.

## Conclusion

Experimental paradigms for BCI and machine learning methods usually are developed and applied independently from each other. In our work, we have shown, how an information theoretical requirement of a decoding approach successfully exerts explicit influence onto the experimental protocol of a BCI paradigm, thus optimizing the interaction of the decoding algorithm, user and paradigm as a whole. We have exemplified this strategy by introducing a novel, easy-to-implement, unsupervised learning approach to the BCI community—learning from label proportions (LLP). Under the assumption of IID data points, the LLP classifier is *guaranteed* to recover class-means of ERP responses, which is not the case for any other unsupervised approach known to the authors.

The experimental protocol of a visual ERP speller was modified by introducing groups of stimulation sequences, with the goal to meet the theoretical requirements of the LLP classifier as good as possible, before we applied it practically in simulations as well as in an online spelling experiment. We found that our protocol adaptation was successful, as the central homogeneity requirement (class-wise means are equal for all stimulation sequences) was violated in only one out of 26 conditions. Even when the violation occurred, the performance was good. Furthermore, we observed that LLP succeeded in estimating the class means from unlabelled data, and found that this classifier works well in practice even though its IID assumption is not realistic for ERP-EEG data.

In the online scenario, 12 out of 13 untrained healthy young participants were able to use the LLP-controlled spelling application without explicit calibration. Comparisons with an EM-based unsupervised classification approach indicate that LLP’s performance on small unlabelled data sets is highly competitive, but that its theoretical guarantees come at the cost of slower convergence for larger number of data.

Future work will investigate the combination of LLP with other unsupervised classification and transfer learning approaches. Additionally, application fields outside of visual ERP paradigms will be explored.

## References

[1] Farwell LA, Donchin E. Talking off the top of your head: toward a mental prosthesis utilizing event-related brain potentials. Electroencephalography and clinical Neurophysiology. 1988;70(6):510–523. 10.1016/0013-4694(88)90149-6 2461285

[2] Schreuder M, Blankertz B, Tangermann M. A new auditory multi-class brain-computer interface paradigm: spatial hearing as an informative cue. PloS one. 2010;5(4):e9813 10.1371/journal.pone.0009813 20368976 PMC2848564

[3] Furdea A, Halder S, Krusienski D, Bross D, Nijboer F, Birbaumer N, et al An auditory oddball (P300) spelling system for brain-computer interfaces. Psychophysiology. 2009;46(3):617–625. 10.1111/j.1469-8986.2008.00783.x 19170946

[4] Simon N, Käthner I, Ruf CA, Pasqualotto E, Kübler A, Halder S. An auditory multiclass brain-computer interface with natural stimuli: Usability evaluation with healthy participants and a motor impaired end user. Frontiers in human neuroscience. 2015;8:1039 10.3389/fnhum.2014.01039 25620924 PMC4288388

[5] Schreuder M, Thurlings ME, Brouwer AM, Van Erp JB, Tangermann M. Exploring the use of tactile feedback in an ERP-based auditory BCI. In: 2012 Annual International Conference of the IEEE Engineering in Medicine and Biology Society. IEEE; 2012. p. 6707–6710. 10.1109/EMBC.2012.6347533 23367468

[6] Brouwer AM, Van Erp JB. A tactile P300 brain-computer interface. Frontiers in neuroscience. 2010;4:19 10.3389/fnins.2010.00019 20582261 PMC2871714

[7] Blankertz B, Tangermann M, Vidaurre C, Fazli S, Sannelli C, Haufe S, et al The Berlin Brain-Computer Interface: Non-Medical Uses of BCI Technology. Frontiers in Neuroscience. 2010;4:198 10.3389/fnins.2010.00198 21165175 PMC3002462

[8] Bensch M, Karim AA, Mellinger J, Hinterberger T, Tangermann M, Bogdan M, et al Nessi: An EEG-Controlled Web Browser for Severely Paralyzed Patients. Computational Intelligence and Neuroscience. 2007;2007:e71863 10.1155/2007/71863 PMC2266985 18350132

[9] Finke A, Lenhardt A, Ritter H. The MindGame: a P300-based brain—computer interface game. Neural Networks. 2009;22(9):1329–1333. 10.1016/j.neunet.2009.07.003 19635654

[10] Congedo M, Goyat M, Tarrin N, Ionescu G, Varnet L, Rivet B, et al. “Brain Invaders”: a prototype of an open-source P300-based video game working with the OpenViBE platform. In: 5th International Brain-Computer Interface Conference 2011 (BCI 2011); 2011. p. 280–283.

[11] Kaplan AY, Shishkin SL, Ganin IP, Basyul IA, Zhigalov AY. Adapting the P300-based brain—computer interface for gaming: a review. IEEE Transactions on Computational Intelligence and AI in Games. 2013;5(2):141–149. 10.1109/TCIAIG.2012.2237517

[12] Tangermann M, Schreuder M, Dähne S, Höhne J, Regler S, Ramsay A, et al Optimized stimulation events for a visual ERP BCI. Int J Bioelectromagn. 2011;13(3):119–120.

[13] Blankertz B, Acqualagna L, Dähne S, Haufe S, Schultze-Kraft M, Sturm I, et al The Berlin Brain-Computer Interface: progress beyond communication and Control. Frontiers in Neuroscience. 2016;10:530 10.3389/fnins.2016.00530 27917107 PMC5116473

[14] Münßinger JI, Halder S, Kleih SC, Furdea A, Raco V, Hösle A, et al Brain painting: first evaluation of a new brain—computer interface application with ALS-patients and healthy volunteers. Frontiers in neuroscience. 2010;4:182 10.3389/fnins.2010.00182 21151375 PMC2996245

[15] Fazel-Rezai R, Allison BZ, Guger C, Sellers EW, Kleih SC, Kübler A. P300 brain computer interface: current challenges and emerging trends. Frontiers in Neuroengineering. 2012;5:14 10.3389/fneng.2012.00014 22822397 PMC3398470

[16] Guger C, Daban S, Sellers E, Holzner C, Krausz G, Carabalona R, et al How many people are able to control a P300-based brain—computer interface (BCI)? Neuroscience letters. 2009;462(1):94–98. 10.1016/j.neulet.2009.06.045 19545601

[17] Sellers EW, Donchin E. A P300-based brain—computer interface: initial tests by ALS patients. Clinical neurophysiology. 2006;117(3):538–548. 10.1016/j.clinph.2005.06.027 16461003

[18] Wolpaw JR, Birbaumer N, McFarland DJ, Pfurtscheller G, Vaughan TM. Brain—computer interfaces for communication and control. Clinical neurophysiology. 2002;113(6):767–791. 10.1016/S1388-2457(02)00057-3 12048038

[19] Shenoy P, Krauledat M, Blankertz B, Rao RP, Müller K-R. Towards adaptive classification for BCI. Journal of neural engineering. 2006;3(1):R13 10.1088/1741-2560/3/1/R02 16510936

[20] Barachant A, Congedo M. A plug&play P300 BCI using information geometry. arXiv preprint arXiv: 14090107. 2014;.

[21] Kindermans PJ, Verstraeten D, Schrauwen B. A Bayesian model for exploiting application constraints to enable unsupervised training of a P300-based BCI. PloS one. 2012;7(4):e33758 10.1371/journal.pone.0033758 22496763 PMC3319551

[22] Kindermans PJ, Schreuder M, Schrauwen B, Müller K-R, Tangermann M. True zero-training brain-computer interfacing—an online study. PloS one. 2014;9(7):e102504 10.1371/journal.pone.0102504 25068464 PMC4113217

[23] Kindermans PJ, Tangermann M, Müller K-R, Schrauwen B. Integrating dynamic stopping, transfer learning and language models in an adaptive zero-training ERP speller. Journal of neural engineering. 2014;11(3):035005 10.1088/1741-2560/11/3/035005 24834896

[24] Vidaurre C, Kawanabe M, von Bünau P, Blankertz B, Müller K-R. Toward unsupervised adaptation of LDA for brain—computer interfaces. IEEE Transactions on Biomedical Engineering. 2011;58(3):587–597. 10.1109/TBME.2010.2093133 21095857

[25] Krauledat M, Tangermann M, Blankertz B, Müller K-R. Towards zero training for brain-computer interfacing. PloS one. 2008;3(8):e2967 10.1371/journal.pone.0002967 18698427 PMC2500157

[26] Fazli S, Dähne S, Samek W, Bießmann F, Müller K-R. Learning from more than one data source: data fusion techniques for sensorimotor rhythm-based Brain-Computer Interfaces. Proceedings of the IEEE. 2015;103(6):891–906. 10.1109/JPROC.2015.2413993.

[27] Fazli S, Popescu F, Danóczy M, Blankertz B, Müller K-R, Grozea C. Subject-independent mental state classification in single trials. Neural networks: the official journal of the International Neural Network Society. 2009;22(9):1305–1312. 10.1016/j.neunet.2009.06.003 19560898

[28] Jayaram V, Alamgir M, Altun Y, Scholkopf B, Grosse-Wentrup M. Transfer learning in brain-computer interfaces. IEEE Computational Intelligence Magazine. 2016;11(1):20–31. 10.1109/MCI.2015.2501545

[29] Lu S, Guan C, Zhang H. Unsupervised brain computer interface based on intersubject information and online adaptation. IEEE Transactions on Neural Systems and Rehabilitation Engineering. 2009;17(2):135–145. 10.1109/TNSRE.2009.2015197 19228561

[30] Quadrianto N, Smola AJ, Caetano TS, Le QV. Estimating labels from label proportions. Journal of Machine Learning Research. 2009;10(Oct):2349–2374.

[31] Patrini G, Nock R, Caetano T, Rivera P. (Almost) no label no cry In: Ghahramani Z, Welling M, Cortes C, Lawrence ND, Weinberger KQ, editors. Advances in Neural Information Processing Systems 27. Curran Associates, Inc.; 2014 p. 190–198. Available from: http://papers.nips.cc/paper/5453-almost-no-label-no-cry.pdf.

[32] Blankertz B, Lemm S, Treder M, Haufe S, Müller K-R. Single-trial analysis and classification of ERP components, A tutorial. Neuroimage. 2011;56(2):814–825. 10.1016/j.neuroimage.2010.06.048 20600976

[33] Verhoeven T, Buteneers P, Wiersema J, Dambre J, Kindermans P. Towards a symbiotic brain—computer interface: exploring the application—decoder interaction. Journal of Neural Engineering. 2015;12(6):066027 10.1088/1741-2560/12/6/066027 26580120

[34] Johannes S, Münte T, Heinze H, Mangun GR. Luminance and spatial attention effects on early visual processing. Cognitive Brain Research. 1995;2(3):189–205. 10.1016/0926-6410(95)90008-X 7580401

[35] Gonsalvez CJ, Polich J. P300 amplitude is determined by target-to-target interval. Psychophysiology. 2002;39(3):388–396. 10.1017/S0048577201393137 12212658

[36] Ledoit O, Wolf M. A well-conditioned estimator for large-dimensional covariance matrices. Journal of multivariate analysis. 2004;88(2):365–411. 10.1016/S0047-259X(03)00096-4

[37] Vidaurre C, Krämer N, Blankertz B, Schlögl A. Time domain parameters as a feature for EEG-based brain—computer interfaces. Neural Networks. 2009;22(9):1313–1319. 10.1016/j.neunet.2009.07.020 19660908

[38] Schäfer J, Strimmer K, et al A shrinkage approach to large-scale covariance matrix estimation and implications for functional genomics. Statistical applications in genetics and molecular biology. 2005;4(1):32. 10.2202/1544-6115.1175 16646851

[39] Bishop CM. Pattern recognition. Machine Learning. 2006;128:1–58.

[40] Thompson DE, Quitadamo LR, Mainardi L, Gao S, Kindermans PJ, Simeral JD, et al Performance measurement for brain—computer or brain—machine interfaces: a tutorial. Journal of neural engineering. 2014;11(3):035001 10.1088/1741-2560/11/3/035001 24838070 PMC4185283

[41] Parra LC, Spence CD, Gerson AD, Sajda P. Recipes for the linear analysis of EEG. Neuroimage. 2005;28(2):326–341. 10.1016/j.neuroimage.2005.05.032 16084117

[42] Chen X, Wang Y, Nakanishi M, Gao X, Jung TP, Gao S. High-speed spelling with a noninvasive brain—computer interface. Proceedings of the national academy of sciences. 2015;112(44):E6058–E6067. 10.1073/pnas.1508080112 PMC4640776 26483479

[43] Jin J, Allison BZ, Wang X, Neuper C. A combined brain—computer interface based on P300 potentials and motion-onset visual evoked potentials. Journal of neuroscience methods. 2012;205(2):265–276. 10.1016/j.jneumeth.2012.01.004 22269596

[44] Townsend G, LaPallo B, Boulay C, Krusienski D, Frye G, Hauser C, et al A novel P300-based brain—computer interface stimulus presentation paradigm: moving beyond rows and columns. Clinical Neurophysiology. 2010;121(7):1109–1120. 10.1016/j.clinph.2010.01.030 20347387 PMC2879474

[45] Treder MS, Blankertz B. (C)overt attention and visual speller design in an ERP-based brain-computer interface. Behavioral and brain functions. 2010;6(1):1 10.1186/1744-9081-6-28 20509913 PMC2904265

[46] Yeom SK, Fazli S, Müller K-R, Lee SW. An efficient ERP-based brain-computer interface using random set presentation and face familiarity. PloS one. 2014;9(11):e111157 10.1371/journal.pone.0111157 25384045 PMC4226481

[47] Kaufmann T, Schulz S, Grünzinger C, Kübler A. Flashing characters with famous faces improves ERP-based brain—computer interface performance. Journal of neural engineering. 2011;8(5):056016 10.1088/1741-2560/8/5/056016 21934188

[48] Hong B, Guo F, Liu T, Gao X, Gao S. N200-speller using motion-onset visual response. Clinical Neurophysiology. 2009;120(9):1658–1666. 10.1016/j.clinph.2009.06.026. 19640783

[49] Tangermann M, Höhne J, Schreuder M, Sagebaum M, Blankertz B, Ramsay A, et al. Data driven neuroergonomic optimization of BCI stimuli. In: Proceedings of 5th International BCI Conference, Graz.; 2011. p. 160–163.

[50] Sugiyama M, Krauledat M, Müller K-R. Covariate shift adaptation by importance weighted cross validation. Journal of Machine Learning Research. 2007;8:1027–1061.

[51] von Bünau P, Meinecke FC, Király F, Müller K-R. Finding stationary subspaces in multivariate time series. Physical Review Letters. 2009;103:214101 10.1103/PhysRevLett.103.214101 20366040

